# ‘What About Swallowing?’ Diagnostic Performance of Daily Clinical Practice Compared with the Eating Assessment Tool-10

**DOI:** 10.1007/s00455-015-9680-8

**Published:** 2016-01-11

**Authors:** Bas Joris Heijnen, Renée Speyer, Margareta Bülow, Laura MF Kuijpers

**Affiliations:** Department of Otorhinolaryngology and Head and Neck Surgery, Leiden University Medical Centre, PO Box 9600, 2300 RC Leiden, The Netherlands; College of Healthcare Sciences, James Cook University, Townsville, QLD Australia; Diagnostic Centre of Imaging and Functional Medicine, Malmö, Sweden; Department of Clinical Sciences, Lund University, Malmö, Sweden; Skane University Hospital Malmö, Malmö, Sweden; Department of Clinical Sciences, Institute of Tropical Medicine, Antwerp, Belgium

**Keywords:** Deglutition, Deglutition disorders, Dysphagia, Swallowing disorders, Screening, Diagnostic performance

## Abstract

In daily clinical practice, patients are frequently asked about their swallowing as part of the patient-clinician interview. This study compares the diagnostic performance of a single open question ‘What about swallowing?’ (usual care) with the Eating Assessment Tool (EAT-10) as reference test in screening for oropharyngeal dysphagia (OD). 303 outpatients at risk of OD were recruited at three university hospitals: 162 men and 141 women with a mean age of 70 years. All data were retrieved by phone. To identify patients at risk of dysphagia, two different cut-off scores for the EAT-10 total score were retrieved from the literature. The diagnostic performance of the single question was determined by comparing dichotomized answers to the single question (no problems versus difficulties in swallowing) with the EAT-10 as reference test. Sensitivity, specificity, positive and negative predictive values ranged between 0.75–0.76, 0.75–0.84, 0.93–0.97 and 0.38–0.43, respectively. Mostly, the results of this exploratory study indicate a sufficient diagnostic performance of the single question in identifying patients who are at risk of dysphagia when using the EAT-10 questionnaire as a reference test. Further research, is, however, necessary to provide additional psychometric data on Functional Health Status (FHS) questionnaires including the single question using either FEES or VFS as gold standard or reference test.

## Introduction

Oropharyngeal dysphagia (OD) is associated with malnutrition, dehydration, aspiration pneumonia, and sudden death [1, 2]. It is known to affect social life [3]: patients may no longer enjoy eating and drinking, and may avoid social activities. OD may, therefore, have a major impact on a patient’s Health-Related Quality of Life (HR-QoL) [2–4].

HR-QoL is the effect of (chronic) medical conditions and their treatment on daily functioning and quality of life (QoL) [5], which is ‘‘a state of complete physical, mental and social well-being, not merely the absence of disease or infirmity’’ [6], as defined by the World Health Organization (WHO) in 1946 [6]. A recent systematic review by Timmerman et al. [7] gives an overview of HR-QoL questionnaires regarding dysphagia. Examples of these questionnaires are the Dysphagia Handicap Index (DHI) [8], the M.D. Andersen Dysphagia Inventory [9] and the SWAL-QOL [10–12].

The gold standard for detecting dysphagia is fibre optic endoscopic evaluation of swallowing (FEES) [13] or video fluoroscopy of the swallowing act (VFS) [13, 14]. The importance of detecting OD at an early stage is being recognized more frequently. Most examinations can, however, be burdensome, time-consuming and costly [15], and therefore, not performed as routine clinical practice in every patient visiting an otorhinolaryngology department.

Another way of screening for OD is the use of a Functional Health Status (FHS) questionnaire, which quantifies the influence of a given disease on particular functional aspects as experienced by the patient [4]. In OD, FHS questionnaires quantify the severity of the swallowing problem [4, 16]. A recent systematic review by Speyer et al. [4] retrieved three English-language questionnaires on FHS in adults with OD: the Eating Assessment Tool (EAT-10) [17], the swallowing outcome after laryngectomy (SOAL) [18], and the Self-report Symptom Inventory. The Sydney Swallow Questionnaire (SSQ) [19] proved to be identical to the Modified Self-report Symptom Inventory.

The Eating Assessment Tool (EAT-10) by Belafsky et al. [17] is a short 10-item, easy to use, self-administered questionnaire [4]. Although the EAT-10 is considered to be predominantly a questionnaire on FHS, some items on HR-QoL are also included. The sum score of this 10-item questionnaire ranges from 0 to 40 [17]. Belafsky et al. [17] found that a sum score ≥3 indicates that a patient is at risk of dysphagia and warrants further examination. In a recent study by Rofes et al. [20], however, it was determined that a cut-off score ≥2 would be optimal. Rofes et al. [20] calculated the sensitivity and specificity of the EAT-10 using VFS as a reference test (golden standard [21]). By a cut-off score of ≥2, the sensitivity and specificity for OD was 89 and 82 %, respectively. Lately Cheney et al. [22] evaluated the ability of the EAT-10 to screen for aspiration risk in patients with dysphagia describing a cut-off score of >15: sensitivity 71 % and specificity 53 %. As Cheney et al. used the EAT-10 not just to screen for OD but to screen for aspiration in selected patients with OD, cut-off points differed highly from earlier data by Belafsky et al. [17] and Rofes et al. [20].

In daily clinical practice, however, a single question such as ‘What about swallowing?’ is frequently used without any additional standardized testing. For example, general practitioners may restrict their patient history on swallowing to a single question, whereas clinicians in specialized dysphagia clinics will include standardized questionnaires such as the EAT-10 as part of the assessment and management of dysphagia. The diagnostic performance of a single question has not been explored until now. If a patient’s answer was negative, it is possible that no further swallowing screening or assessment would be performed. As symptoms like coughing, choking, feeling the food sticking (in the throat) after swallowing and respiration problems may all be aspects of OD, a single question might expect a patient to have preliminary knowledge about the concept of dysphagia. Therefore, the use of a single question on swallowing instead of a more detailed questionnaire such as the EAT-10, might lead to an under-diagnosis of those patients at risk of dysphagia.

The purpose of the current study is to compare the diagnostic performance of a single question on swallowing (usual care) with the FHS questionnaire EAT-10 as reference test. Two different EAT-10 cut-off scores for patients at risk of dysphagia will be used: a sum score ≥3 as suggested by Belafsky et al. [17] and ≥2 as defined by Rofes et al. [20]. We hypothesize that a single question, ‘What about swallowing?’, which is part of everyday clinical practice, will show poor diagnostic performance when compared to the EAT-10. It is expected that the single question will have insufficient sensitivity and specificity to identify patients at risk of dysphagia.

## Methods

### Subjects

We studied a consecutive series of new patients who visited the outpatient clinics for dysphagia or otorhinolaryngology of the Leiden University Medical Centre (LUMC), Maastricht University Medical Centre (MUMC) and Skane University Hospital Malmö (SUS Malmö). Included were participants aged at least 18 years of age who might be at risk of OD. Patients with severe cognitive problems were excluded. Within 6 months of their initial visit to the clinics, patients were contacted by telephone. All data were collected during that call.

### Protocol

First, patients were invited to participate when contacted by phone. After informed consent and during that same phone call, data on the current status of the patients were collected. Subject characteristics including age, gender and actual oral intake were registered. The latter was assessed using the functional oral intake scale (FOIS) which ranges from 1 (i.e. nothing by mouth) to 7 (i.e. no restrictions) [23]. Subsequently, a single question was posed, representing clinical daily practice: ‘What about swallowing?’. All answers were written down and at a later stage dichotomized, to normal (i.e. no complaints) and abnormal (i.e. at least mild complaints). For example, participants responded ‘I can eat and drink everything’ (normal) or ‘Sometimes meat gets stuck in my throat’ (abnormal). Finally, the EAT-10 was administered. The EAT-10 consists of ten questions which can be scored from 0 (no problem) to 4 (severe problem). The range of the sum score is 0–40 [17].

### Statistics

Apart from descriptive data analysis, the sensitivity, specificity, positive predictive value (PPV) and negative predictive value (NPV) of the single question ‘What about swallowing?’ were calculated. The EAT-10 was used as a reference test. A sensitivity of ≥70 % and a specificity of ≥60 % was considered as minimum requirement for screening instruments [24]. Both cut-off scores by Belafsky et al. [17] and Rofes et al. [20] were used to identify patients at risk of dysphagia.

## Results

The LUMC, MUMC and SUS Malmö included 303 patients (78, 122 and 103 patients, respectively). Patient characteristics are provided in Table 1. One hundred and sixty-two patients (53 %) were male with a median age of 70 years (IQR, 60–77 years), and 141 were female with a median age of 69 years (IQR, 57–76 years). Medical diagnoses included head and neck cancer (15 %) and neurological diseases such as stroke, Parkinson’s disease, multiple sclerosis or myotonic dystrophy (46 %). A third group of patients suffered from a variety of diseases such as general weakness due to other diseases, cricopharyngeus hypertrophia, epiglottitis, etc (39 %). Most patients followed an oral intake without any restrictions: The median FOIS score was 7 (IQR, 5–7).

**Table 1:** Subject characteristics (number of subjects, gender, age, FOIS and medical diagnoses per center

Subject characteristic	Patient recruitment (centre)			Total
	LUMC	SUS Malmö	MUMC	
Number of subjects	78	103	122	303
Gender (M;F)	34 M; 44 F	50 M; 53 F	78 M; 44 F	162 M; 141 F
Age in years (Med; IQR)				
All	67; 53–76	74; 64–79	69; 62–74	70; 60–77
Male	67; 56–71	75; 66–79	69; 64–75	70; 63–77
Female	69; 50–76	73; 62–79	68; 55–73	69; 57–76
FOIS (Med; IQR)	7; 6–7	6; 5–7	6; 5–7	7; 5–7
Medical diagnoses (*N*; %)				
Head and neck cancer	27; 35	3; 3	16; 13	46; 15
Neurological disorder	16; 20	28; 27	95; 78	139; 46
Other	35; 45	72; 70	11; 9	118; 39

**Fig. 1:**
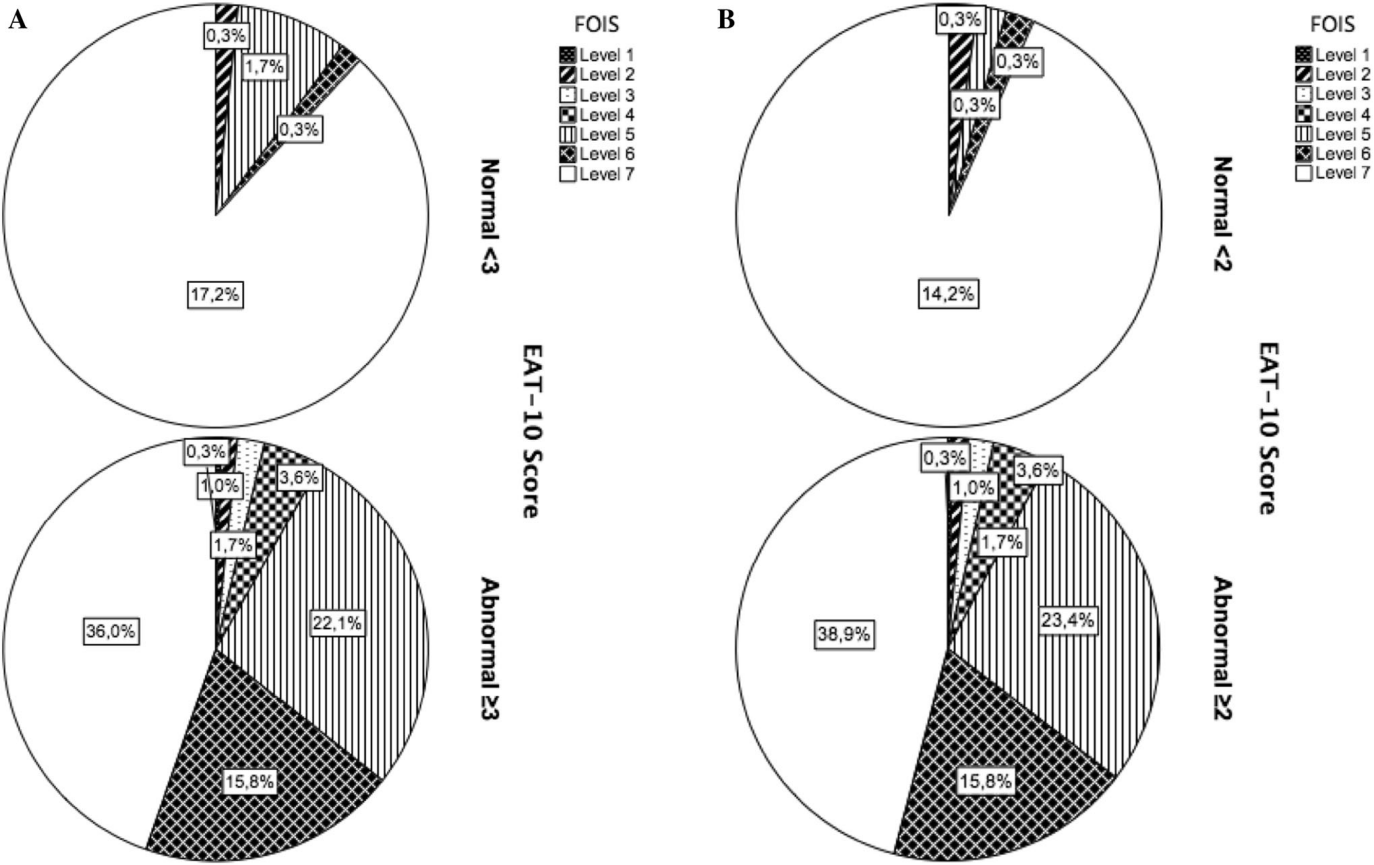
**a** FOIS levels in percentages by dichotomized EAT-10 scores (cut-off score ≥3) [17]. **b** FOIS levels in percentages by dichotomized EAT-10 scores (cut-off score ≥2) [20]

Figure 1A shows the FOIS levels in relation to the dichotomized EAT-10 scores using a cut-off score of ≥3 points according to Belafsky et al. [11]. to distinguish patients at risk of dysphagia and those demonstrating normal swallowing. The data in the figure indicates that 36.0 % of the total population obtained an abnormal EAT-10 score, thus being at risk of dysphagia, while oral intake was normal, whereas 2.3 % of the total population obtained a normal EAT-10 score while their oral intake was restricted. This may suggest that a cut-off point of ≥3 misses 2.3 % of participants who seem at risk of dysphagia. When using the cut-off score of ≥2 points by Rofes et al. [20], the following data are found (see Fig. 1b): 38.9 % of the total population producing an abnormal EAT-10 score have a normal oral intake, whereas 1.0 % of those with a normal EAT-10 score have an oral intake with restrictions.

**Fig. 2:**
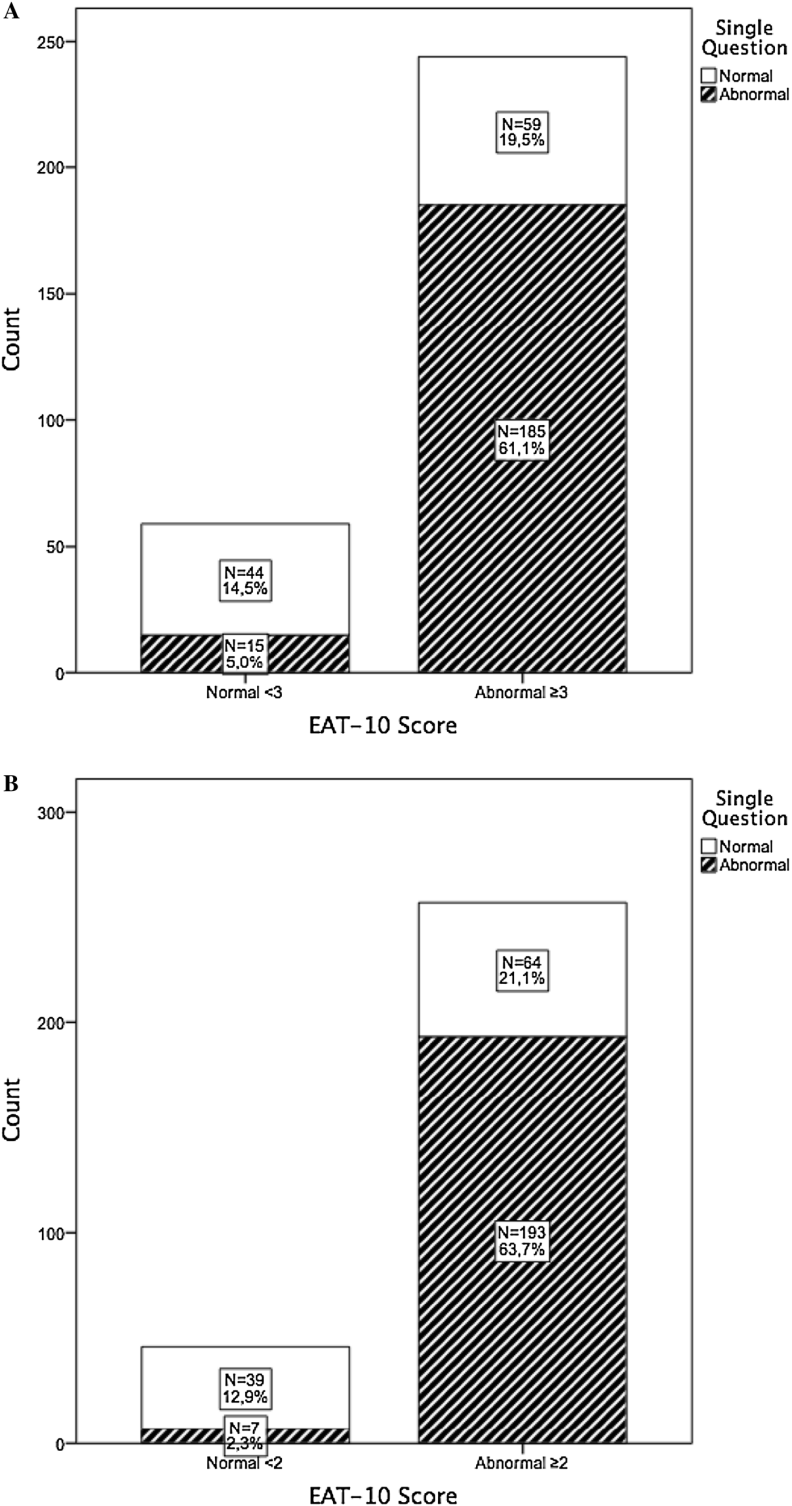
**a** Subjects at risk of OD: Data on single question by dichotomized EAT-10 scores (cut-off score ≥3) [17]. **b** Subjects at risk of OD: Data on single question by dichotomized EAT-10 scores (cut-off score ≥2) [20]

Figure 2 A displays the answer to the single question ‘What about swallowing?’ in relation to the EAT-10 outcome using the cut-off score by Belafsky and underlines the previous findings shown in Fig. 1a. A total of 200 (66.1 %) patients report having swallowing problems when answering the single question. Two-hundred and forty-four of these patients were at risk of dysphagia according to the EAT-10. In 103 patients (34.0 %) the single question was scored as normal; however, 59 (19.5 %) of these patients were at risk of dysphagia according to the EAT-10. Figure 2b shows similar data using the cut-off score by Rofes et al. [20]. In 103 patients (34 %), the single question was scored as normal; however, 64 (21.1 %) of these patients were at risk of dysphagia according to the EAT-10.

**Fig. 3:**
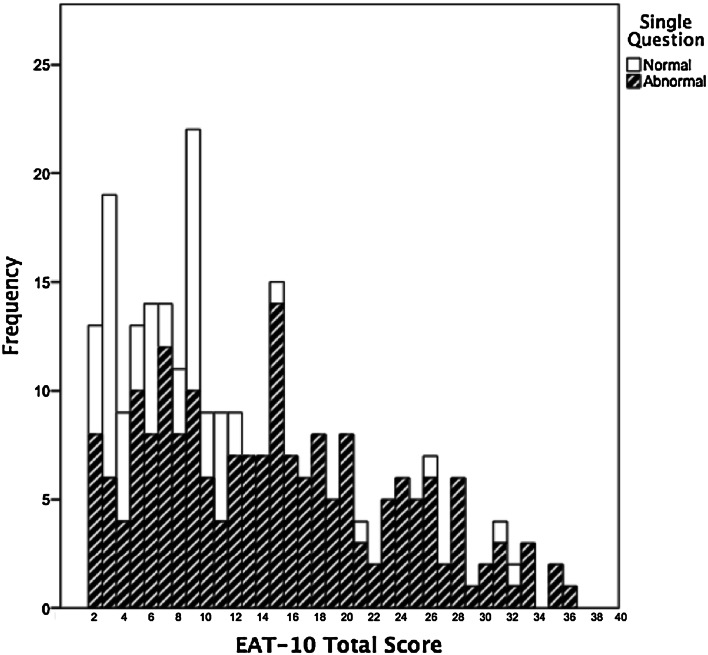
Distribution of data on single question by EAT-10 total score

In Fig. 3, the distribution is displayed of the answers to the single question versus the EAT-10 total score. The histogram shows that the patients who report having no swallowing problem on the single question can score ≥3 points on the EAT-10, with some patients having EAT-10 sum scores up to 32.

**Fig. 4:**
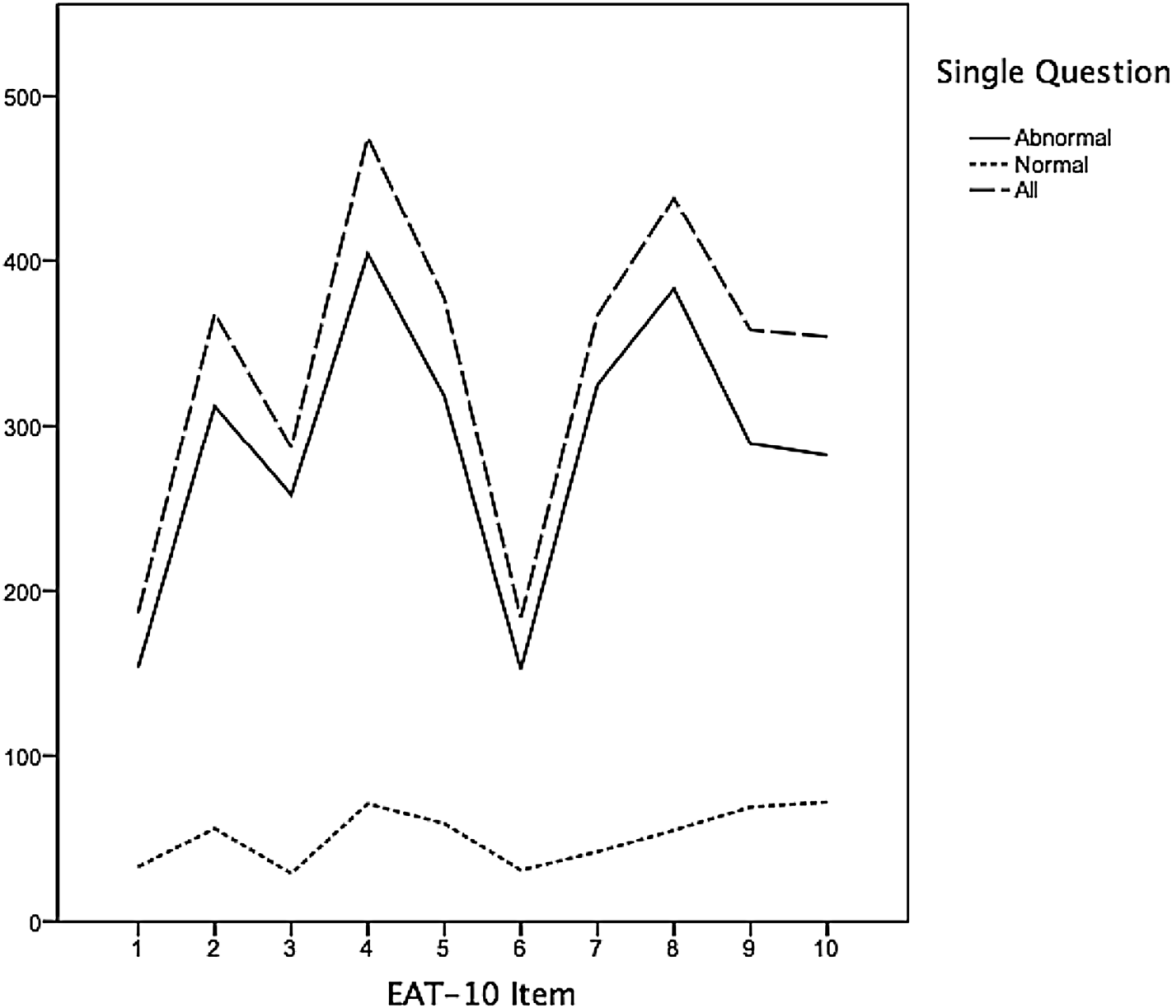
Sum of all total scores per EAT-10 item for all subjects (*N* = 303) and subjects with abnormal swallowing (*N* = 200) and normal swallowing (*N* = 103) according to the single question

Table 2 shows the frequencies of the EAT-10 scores per item for three groups: all participants (*N* = 303), subjects with normal swallowing (*N* = 103) and those with abnormal swallowing (*N* = 200) according to the single question. In addition, Fig. 4 illustrates the sum of all total scores per EAT-10 item for the same three groups; higher scores were obtained for items 2, 4 and 8 and lower scores for items 1 and 6. All three groups showed similar tendencies.

**Table 2:** Frequencies of the EAT-10 scores per item for three groups: all participants (*N* = 303), subjects with normal swallowing (*N* = 103) and abnormal swallowing (*N* = 200) according to the single question

EAT-10	Group	EAT-10 item score				
		0 (no problem)	1	2	3	4 (severe problem)
1. My swallowing problem has caused me to lose weight	All (*N* = 303)	219(72.2 %)	28(9.2 %)	28(9.2 %)	13(4.2 %)	16(5.2 %)
	Single question normal (*N* = 103)	82(79.7 %)	13(12.6 %)	6(5.8 %)	0(0 %)	2(1.9 %)
	Single question abnormal (*N* = 200)	136(68 %)	15(7.5 %)	22(11 %)	13(6.5 %)	14(7 %)
2. My swallowing problem interferes with my ability to go out for meals	All (*N* = 303)	161(53.0 %)	35(11.5 %)	30(9.9 %)	39(12.8 %)	39(12.8 %)
	Single question normal (*N* = 103)	75(72.8 %)	14(13.6 %)	4(3.9 %)	6(5.8 %)	4(3.9 %)
	Single question abnormal (*N* = 200)	85(42.5 %)	21(10.5 %)	26(13.0 %)	33(16.5 %)	35(17.5 %)
3. Swallowing liquids takes extra effort	All (*N* = 303)	167(55.0 %)	50(16.5 %)	35(11.4 %)	41(13.5 %)	11(3.6 %)
	Single question normal (*N* = 103)	86(83.5 %)	9(8.7 %)	4(3.9 %)	4(3.9 %)	0(0 %)
	Single question abnormal (*N* = 200)	80(40.0 %)	41(20.5 %)	31(15.5 %)	37(18.5 %)	11(5.5 %)
4. Swallowing solids takes extra effort	All (*N* = 303)	106(34.8 %)	52(17.1 %)	45(14.8 %)	71(23.4 %)	30(9.9 %)
	Single question normal (*N* = 103)	64(62.1 %)	20(19.5 %)	8(7.8 %)	9(8.7 %)	2(1.9 %)
	Single question abnormal (*N* = 200)	41(20.5 %)	32(16.0 %)	37(18.5 %)	62(31.0 %)	28(14.0 %)
5. Swallowing pills takes extra effort	All (*N* = 303)	146(48.1 %)	39(12.8 %)	51(16.8 %)	36(11.8 %)	32(10.5 %)
	Single question normal (*N* = 103)	69(67.0 %)	15(14.6 %)	15(14.6 %)	2(1.9 %)	2(1.9 %)
	Single question abnormal (*N* = 200)	76(38.0 %)	24(12.0 %)	36(18.0 %)	34(17.0 %)	30(15.0 %)
6. Swallowing is painful	All (*N* = 303)	220(72.6 %)	32(10.5 %)	19(6.2 %)	18(5.9 %)	15(4.8 %)
	Single question normal (*N* = 103)	85(82.6 %)	8(7.7 %)	8(7.7 %)	1(1.0 %)	1(1.0 %)
	Single question abnormal (*N* = 200)	134(67.0 %)	24(12.0 %)	11(5.5 %)	17(8.5 %)	14(7.0 %)
7. The pleasure of eating is affected by my swallowing	All (*N* = 303)	155(51.1 %)	35(11.5 %)	43(14.1 %)	38(12.5 %)	33(10.8 %)
	Single question normal (*N* = 103)	81(78.7 %)	11(10.7 %)	4(3.9 %)	5(4.8 %)	2(1.9 %)
	Single question abnormal (*N* = 200)	73(36.5 %)	24(12.0 %)	39(19.5 %)	33(16.5 %)	31(15.5 %)
8. When I swallow food sticks in my throat	All (*N* = 303)	120(39.6 %)	49(16,1 %)	51(16.7 %)	49(16.1 %)	35(11.5 %)
	Single question normal (*N* = 103)	68(66.0 %)	22(21.4 %)	9(8.7 %)	1(1.0 %)	3(2.9 %)
	Single Question abnormal (*N* = 200)	51(25.5 %)	27(13.5 %)	42(21.0 %)	48(24.0 %)	32(16.0 %)
9. I cough when I eat	All (*N* = 303)	138(45.5 %)	55(18.0 %)	51(16.8 %)	39(12.8 %)	21(6.9 %)
	Single question normal (*N* = 103)	59(57.3 %)	26(25.2 %)	13(12.6 %)	3(3.0 %)	2(1.9 %)
	Single question abnormal (*N* = 200)	78(39.0 %)	29(14.5 %)	38(19.0 %)	36(18.0 %)	19(9.5 %)
10. Swallowing is stressful	All (*N* = 303)	148(48.7 %)	45(14.8 %)	47(15.5 %)	41(13.5 %)	23(7.5 %)
	Single question normal (*N* = 103)	66(64.0 %)	16(15.6 %)	10(9.7 %)	8(7.8 %)	3(2.9 %)
	Single question abnormal (*N* = 200)	81(40.5 %)	29(14.5 %)	37(18.5 %)	33(16.5 %)	20(10.0 %)

The diagnostic performance of the single question was determined using the EAT-10 as reference test and the single question as index test (Table 3). Tables 3 and 4 present cross-tabs based on the cut-off sum score according to Belafsky et al. [17] and Rofes et al. [20], respectively. Using a cut-off score of ≥3, the following data are found: sensitivity of 76 %, specificity of 75 %, PPV of 93 % and NPV of 43 % (Table 3). Changing the EAT-10 cut-off score to ≥2 points increases specificity to 84 % and PPV to 97 %, and decreases the sensitivity to 75 % and NPV to 38 % (Table 4).

**Table 3:** Cross-tabs of the EAT-10 using a cut-off score of ≥3 [11] *(Reference test)* and the single question “What about swallowing?” (*Index test*)

	EAT-10 (*Reference test*)		Total
	+ (at risk of OD)	− (not at risk)	
Single question ‘What about swallowing?’ (*Index test*)			
+ (Abnormal)	185	15	200
− (Normal)	59	44	103
Total	244	59	303

Diagnostic performance of the single question: Se = 0.76, Sp = 0.75, PPV = 0.93 and NPV = 0.43

**Table 4:** Cross-tabs of the EAT-10 using a cut-off score of ≥2 [12] (*Reference test*) and the single question (*Index test*)

	EAT-10 (Reference test)		Total
	+ (at risk of OD)	− (not at risk)	
Single question ‘What about swallowing?’ (*Index test*)			
+ (Abnormal)	193	7	200
− (Normal)	64	39	103
Total	257	46	303

Diagnostic performance of the single question: Se = 0.75, Sp = 0.84, PPV = 0.97 and NPV = 0.38

## Discussion

The purpose of the current study was to compare the diagnostic performance of a single question on swallowing with the FHS questionnaire EAT-10 as a reference test to identify patients who are at risk of dysphagia. Although it may be hypothesized that a validated questionnaire may have a higher sensitivity and specificity, a single question is still part of everyday clinical practice and, therefore, its diagnostic performance should be known. For example, most general practitioners may restrict their patient history on swallowing to a single question, whereas clinical experts in OD will ask for more detailed information and will usually include standardized assessments on OD such as the EAT-10.

The use of a measurement tool in clinical practice can only be justified by its validity and reliability. When validating questionnaires, different psychometric characteristics should be taken in account as shown by Terwee et al. [25] and Aaronson et al. [26], such as content validity, internal consistency, criterion validity, construct validity, reproducibility, responsiveness, floor and ceiling effects, and interpretability. In 2010, Mokkink et al. [27] published the Consensus-based Standards for the selection of health Measurement Instruments (COSMIN) [28]: a taxonomy of measurement properties and definitions for health-related patient reported outcomes.

In a psychometric review by Speyer et al. [4] on FHS in OD, three FHS questionnaires were retrieved whose measurement properties were determined using the COSMIN checklist [29] and the 4-point rating scale according to Terwee et al. [30]. All three FHS questionnaires obtained poor overall methodological quality scores for most psychometric properties and, therefore, psychometric re-assessment of all FHS questionnaires was advised. In a more recent publication, Rofes et al. [20] provided additional information on the diagnostic performance of the EAT-10 compared with VFS. The EAT-10 showed an ROC AUC of 0.89 for OD with an optimal cut-off score at two instead of the proposed cut-off at three by Belafsky et al. [17]. The sensitivity and specificity were 0.89 and 0.82, respectively.

In this study, we demonstrated that a single question has sufficient sensitivity and specificity to screen for patients at risk of dysphagia when using the EAT-10 as reference test; depending on the EAT-10 cut-off point, sensitivity and specificity of the single question ranged between 75–76 and 75–84 %, respectively. These values fall within the minimum norms for sensitivity and specificity of ≥70 % and ≥60 % as suggested by Bours et al. [21] or Kertscher et al. [24]. This leads to the rejection of our initial hypothesis that a single question ‘What about swallowing?’ would show poor diagnostic performance when compared to the EAT-10.

However, despite of the sufficient sensitivity and specificity, the low NPV of the single question (ranging between 0.38 and 0.43 depending on the cut-off point) remains a concern and may restrict the use of the single question in screening for dysphagia; a high percentage of subjects (false negatives) will not be considered for further dysphagia assessment even though they are actually at risk for dysphagia. In contrast to the NPV, the PPV (ranging between 0.93 and 0.97) is adequate and only very few subjects (false positives) will be referred for further assessment while not being at risk for dysphagia.

Some methodological remarks can be made, however. First of all, in this study, a Swedish and Dutch consensus translation by dysphagia experts of the EAT-10 was used. These translations were not validated. Furthermore, all data were gathered by phone, whereas the EAT-10 was developed as a patient self-report. Another aspect is the possible priming of patients using a standardized protocol order: the single question was asked first, directly followed by the EAT-10. Finally, the subject population in general showed limited restrictions in oral intake as measured by FOIS, indicating a mild severity of OD. It cannot be ruled out that in the case of patients with more severe swallowing problems, data might have been slightly different from those presented in this manuscript. In our opinion, however, none of these matters is expected to be of significant influence on the reported outcome.

Nonetheless, even though the single open question showed sufficient diagnostic performance, the use of a standardized questionnaire may have advantages. Using a standardized set of questions warrants the retrieval of similar information from all patients and prohibits the omission of essential information. Furthermore, in contrast to the single question, patients do not need to have preliminary knowledge about the concept of dysphagia. A questionnaire could list all associated issues such as coughing, history of pneumonia, etc. Still, in case of the availability of multiple screening tools with sufficient diagnostic performance, different clinical work settings may require different screening tools depending on factors such as number of trained staff, work-load per staff member, availability of FEES or VFS in the setting itself, and possible time constraints [24].

Currently, research is being carried out to determine the diagnostic performance of FHS questionnaires including the single question using either FEES or VFS as reference test. This study will provide additional psychometric data on FHS questionnaires as a screening instrument for patients at risk of OD and the validity and reliability of a single question representing daily clinical practice.

## Conclusion

Because OD is associated with malnutrition, dehydration, aspiration pneumonia, sudden death [1, 2], decreased HR-QoL [7] and is often a complication of other medical problems [31], early detection and adequate screening are important. A single open question ‘What about swallowing?’ is often part of daily clinical practice.

Even though the NPV was rather low, this study found high sensitivity, specificity and PPV data for this single question in identifying patients who are at risk of dysphagia when using the EAT-10 questionnaire as a reference test. Ongoing research will provide additional psychometric data on FHS questionnaires such as the single question using either FEES or VFS as gold standard or reference test. Once the measurement properties of all FHS questionnaires, including daily clinical practice or the single open question, are known, an optimal choice between FHS questionnaires can be justified.

## References

[1] Garcia-Peris P (2007). Long-term prevalence of oropharyngeal dysphagia in head and neck cancer patients: impact on quality of life. Clin Nutr.

[2] Martino R (2005). Dysphagia after stroke: incidence, diagnosis, and pulmonary complications. Stroke.

[3] Eslick GD, Talley NJ (2008). Dysphagia: epidemiology, risk factors and impact on quality of life–a population-based study. Aliment Pharmacol Ther.

[4] Speyer R (2014). Psychometric properties of questionnaires on functional health status in oropharyngeal dysphagia: a systematic literature review. Biomed Res Int.

[5] Wilson IB, Cleary PD (1995). Linking clinical variables with health-related quality of life. A conceptual model of patient outcomes. JAMA.

[6] International Health (2002). C., Constitution of the World Health Organization. 1946. Bull World Health Organ.

[7] Timmerman AA (2014). Psychometric characteristics of health-related quality-of-life questionnaires in oropharyngeal dysphagia. Dysphagia.

[8] Silbergleit AK (2012). The Dysphagia handicap index: development and validation. Dysphagia.

[9] Chen AY (2001). The development and validation of a dysphagia-specific quality-of-life questionnaire for patients with head and neck cancer: the M. D. Anderson dysphagia inventory. Arch Otolaryngol Head Neck Surg.

[10] McHorney CA (2000). The SWAL-QOL outcomes tool for oropharyngeal dysphagia in adults: I. Conceptual foundation and item development. Dysphagia.

[11] McHorney CA (2000). The SWAL-QOL outcomes tool for oropharyngeal dysphagia in adults: II. Item reduction and preliminary scaling. Dysphagia.

[12] McHorney CA (2002). The SWAL-QOL and SWAL-CARE outcomes tool for oropharyngeal dysphagia in adults: III. Documentation of reliability and validity. Dysphagia.

[13] Speyer R (2013). Oropharyngeal dysphagia: screening and assessment. Otolaryngol Clin North Am.

[14] Brady S, Donzelli J (2013). The modified barium swallow and the functional endoscopic evaluation of swallowing. Otolaryngol Clin North Am.

[15] Wilson RD, Howe EC (2012). A cost-effectiveness analysis of screening methods for dysphagia after stroke. PM R.

[16] Ferrans CE (2005). Conceptual model of health-related quality of life. J Nurs Scholarsh.

[17] Belafsky PC (2008). Validity and reliability of the Eating Assessment Tool (EAT-10). Ann Otol Rhinol Laryngol.

[18] Govender R (2012). Development and preliminary validation of a patient-reported outcome measure for swallowing after total laryngectomy (SOAL questionnaire). Clin Otolaryngol.

[19] Wallace KL, Middleton S, Cook IJ (2000). Development and validation of a self-report symptom inventory to assess the severity of oral-pharyngeal dysphagia. Gastroenterology.

[20] Rofes L (2014). Sensitivity and specificity of the Eating Assessment Tool and the Volume-Viscosity Swallow Test for clinical evaluation of oropharyngeal dysphagia. Neurogastroenterol Motil.

[21] Bours GJ (2009). Bedside screening tests vs. videofluoroscopy or fibreoptic endoscopic evaluation of swallowing to detect dysphagia in patients with neurological disorders: systematic review. J Adv Nurs.

[22] Cheney DM (2015). The Ability of the 10-Item Eating Assessment Tool (EAT-10) to Predict Aspiration Risk in Persons With Dysphagia. Ann Otol Rhinol Laryngol.

[23] Crary MA, Mann GD, Groher ME (2005). Initial psychometric assessment of a functional oral intake scale for dysphagia in stroke patients. Arch Phys Med Rehabil.

[24] Kertscher B (2014). Bedside screening to detect oropharyngeal dysphagia in patients with neurological disorders: an updated systematic review. Dysphagia.

[25] Terwee CB (2007). Quality criteria were proposed for measurement properties of health status questionnaires. J Clin Epidemiol.

[26] Aaronson N (2002). Assessing health status and quality-of-life instruments: attributes and review criteria. Qual Life Res.

[27] Mokkink LB (2010). The COSMIN study reached international consensus on taxonomy, terminology, and definitions of measurement properties for health-related patient-reported outcomes. J Clin Epidemiol.

[28] Angst F. The new COSMIN guidelines confront traditional concepts of responsiveness. BMC Med Res Methodol. 2011;11:152 author reply 152. 10.1186/1471-2288-11-152 PMC3231875 22099330

[29] Mokkink LB (2010). The COSMIN checklist for assessing the methodological quality of studies on measurement properties of health status measurement instruments: an international Delphi study. Qual Life Res.

[30] Terwee CB (2012). Rating the methodological quality in systematic reviews of studies on measurement properties: a scoring system for the COSMIN checklist. Qual Life Res.

[31] Roden DF, Altman KW (2013). Causes of dysphagia among different age groups: a systematic review of the literature. Otolaryngol Clin North Am.

